# Supplementary material for: Comparative genome analysis provides a foundation for defining salvinorin A biosynthesis in Salvia divinorum
Source: Nat Commun. 2026 Mar 23;17:3414. doi: 10.1038/s41467-026-70885-3 (PMC13069086; doi:10.1038/s41467-026-70885-3)
Supplement: Supplementary file 1 — Supplementary Information [file 41467_2026_70885_MOESM1_ESM.pdf]

Comparative genome analysis provides a foundation for defining salvinorin A biosynthesis in *Salvia divinorum*

Li and Sun *et al.*

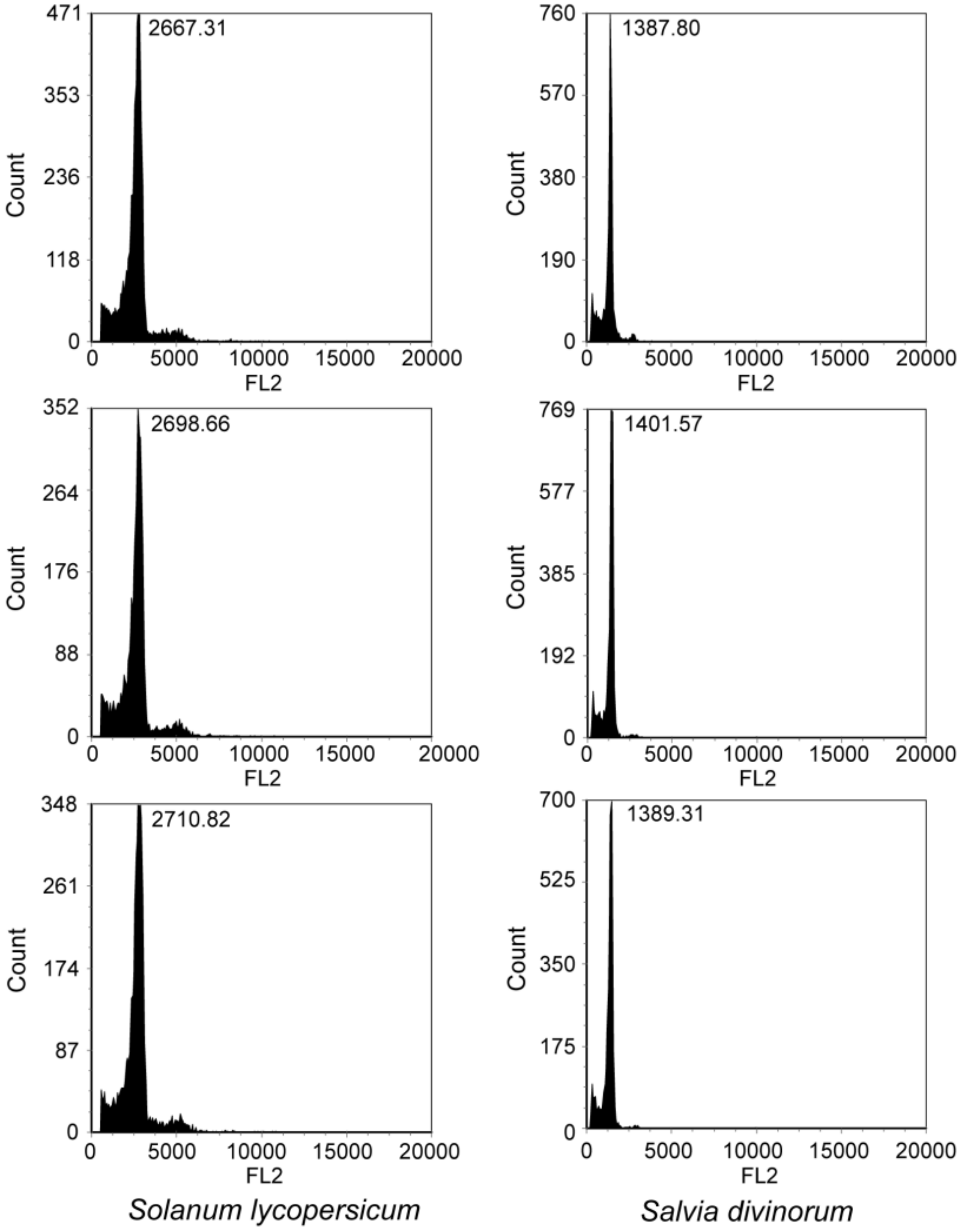

Supplementary Figure 1. ***S. divinorum*** **genome size predicted by Flow Cytometry fluorescene intensity.** *Solanum lycopersicum* was used as the standard/reference. FL2 refers to the fluorescent detection channel for flow cytometry, the FL2 channel detects PI (propidium iodide) fluorescence. Three replicate assays for each species are shown. Source data are provided as a Source Data file.

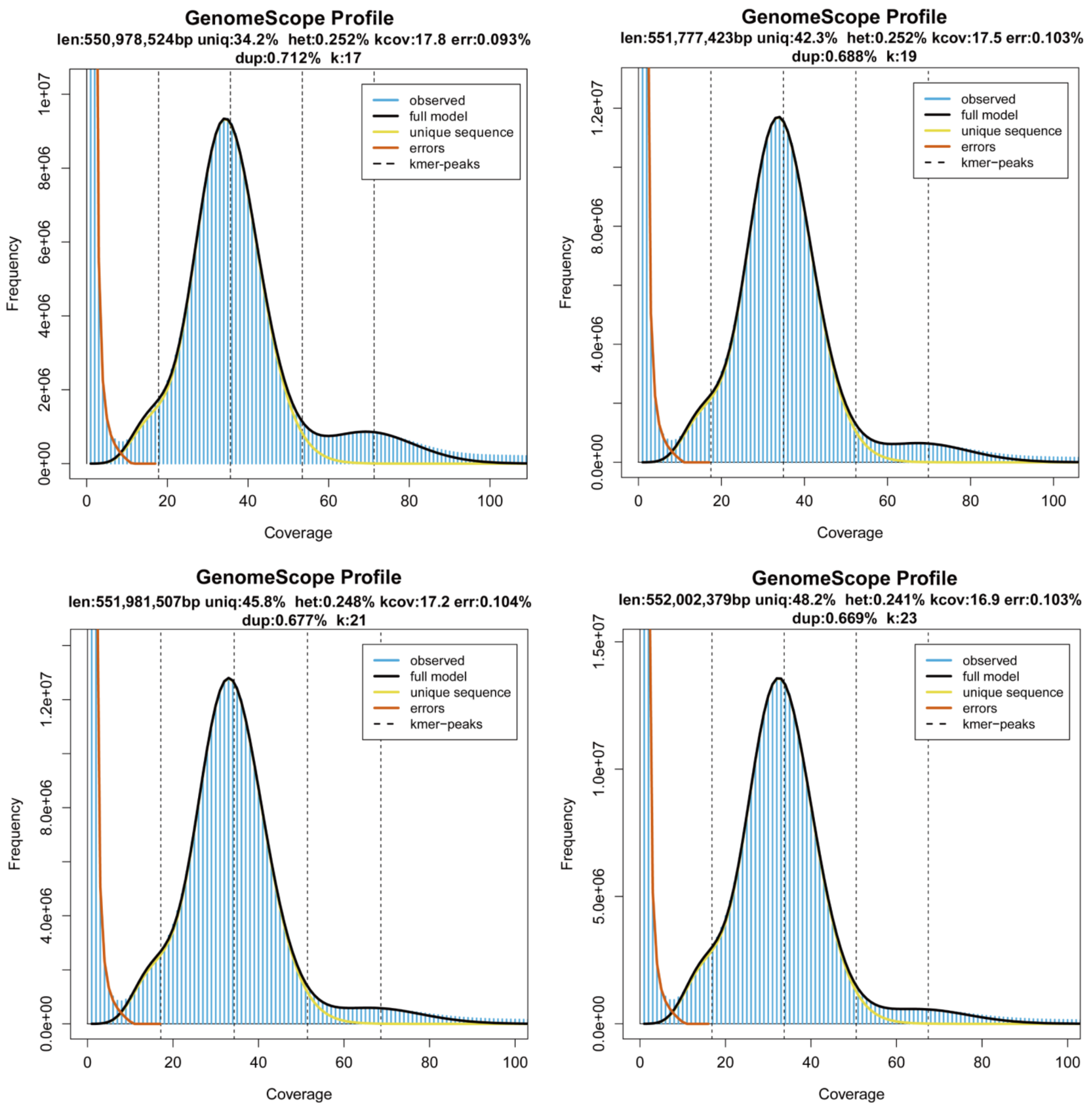

Supplementary Figure 2. Genome size evaluation of *S. divinorum* conducted by k-mer analysis using GenomeScope1. The k-mer distribution assessment reveals revealing a primary peak that signified the homozygous regions of the *S. divinorum* genome. A small shoulder to the left of this peak indicated heterozygous areas, giving a heterozygosity rate of 0.252%. The data by panel reflect the 17-mer, 19-mer, 21-mer, and 23-mer distribution, respectively; the full model trace represents a mixture model that omits presumptive sequencing errors; unique sequences are those without high-frequency repeats; errors refer to sequencing mistakes detected by low coverage k-mers; k-mer peaks consist of four genome components: heterozygous unique regions, homozygous unique regions, heterozygous repetitive regions, and homozygous repetitive regions; Len denotes the estimated total genome length; Het indicates the overall heterozygosity rate. Source data are provided as a Source Data file.

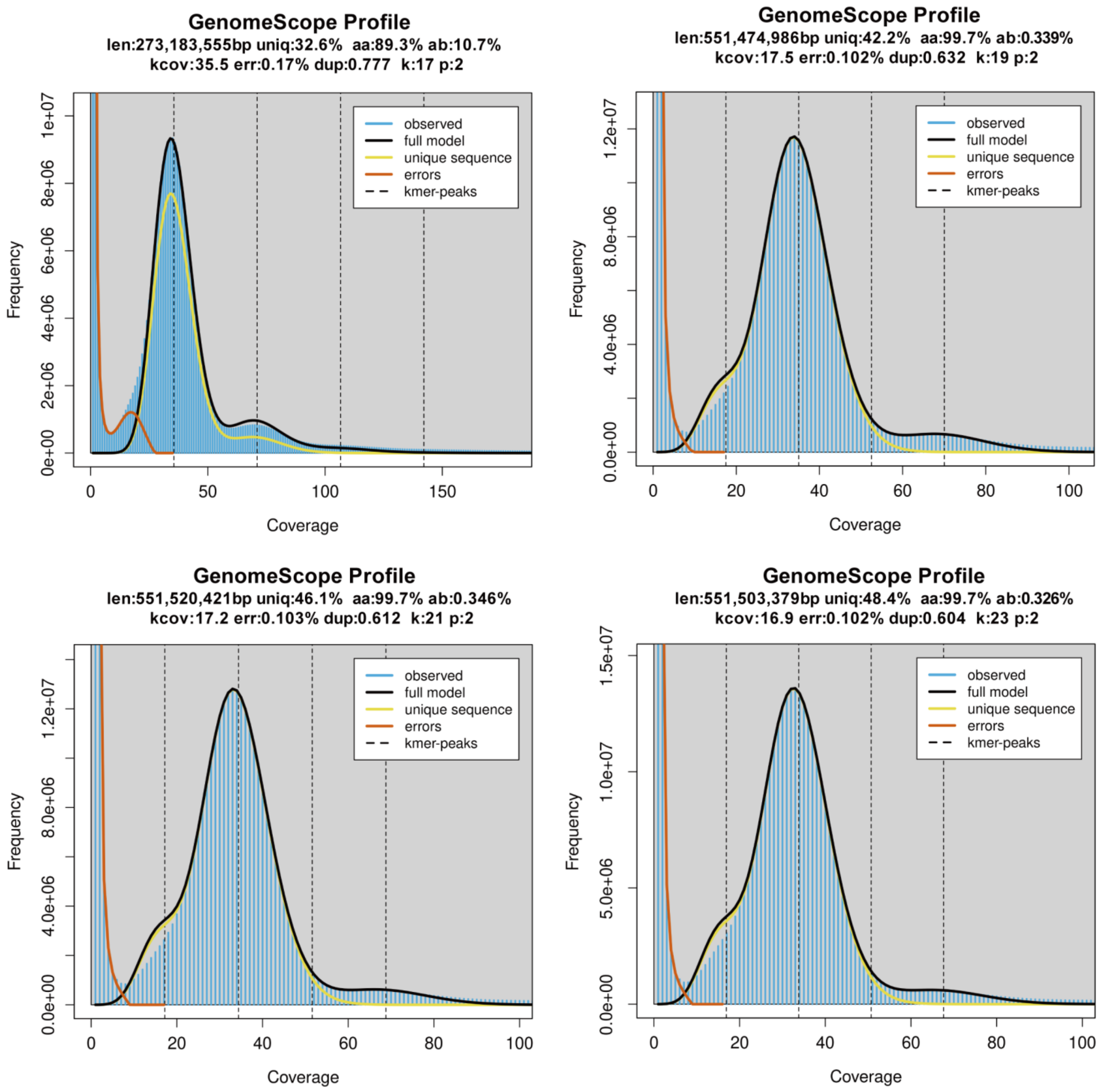

Supplementary Figure 3. Genome size evaluation of *S. divinorum* was conducted by k-mer analysis. using GenomeScope2. The k-mer distribution assessment reveals revealing a primary peak that signified the homozygous regions of the *S. divinorum* genome. A small shoulder to the left of this peak indicated heterozygous areas, giving a heterozygosity rate of 0.252%. The data by each column reflect the 17-mer, 19-mer, 21-mer, and 23-mer distribution, respectively; the full model trace represents a mixture model that omits presumptive sequencing errors; unique sequences are those without high-frequency repeats; errors refer to sequencing mistakes detected by low coverage k-mers; k-mer peaks consist of four genome components: heterozygous unique regions, homozygous unique regions, heterozygous repetitive regions, and homozygous repetitive regions; Len denotes the estimated total genome length; Het indicates the overall heterozygosity rate. Source data are provided as a Source Data file.

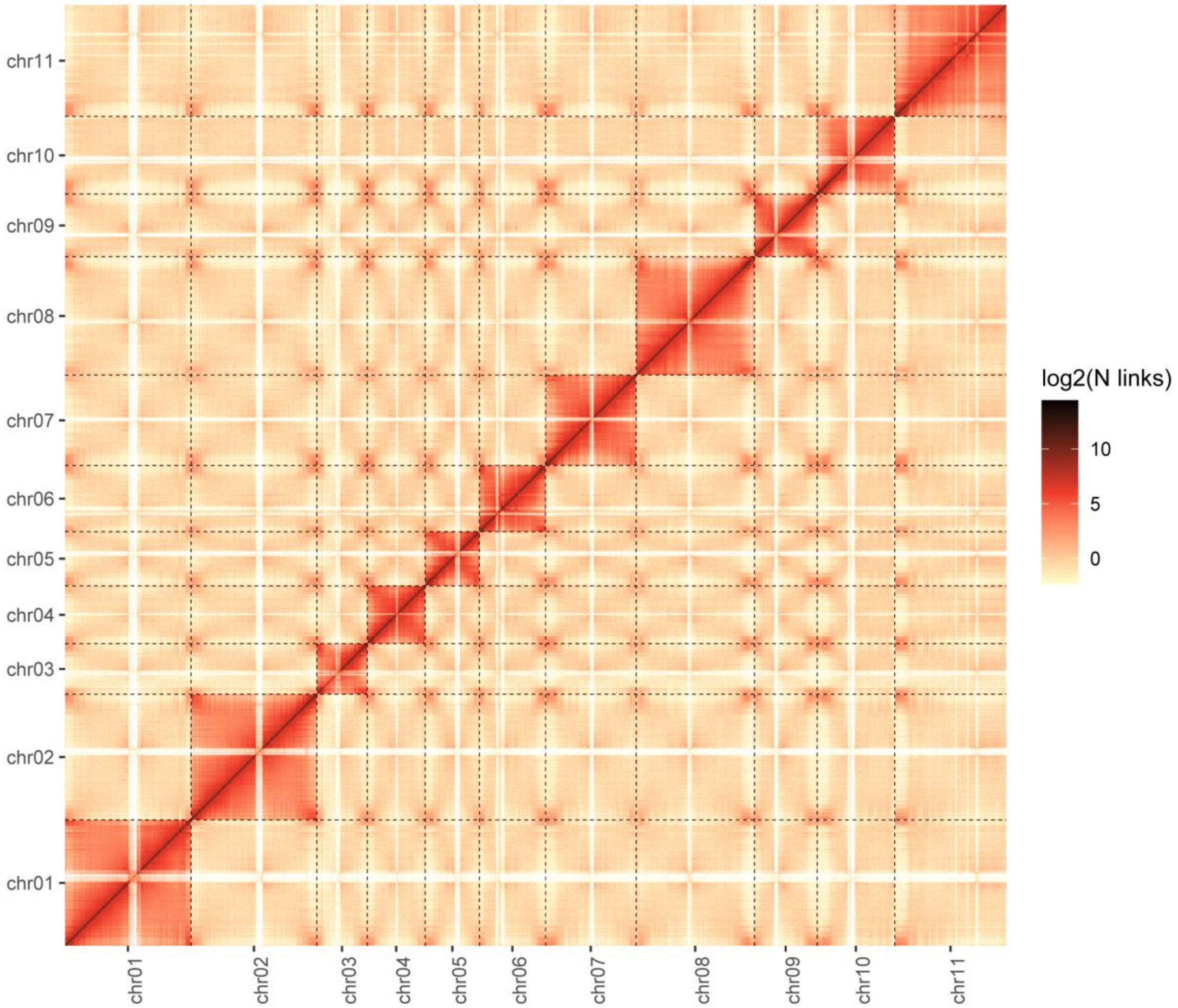

Supplementary Figure 4. Hi-C contact frequencies for *S. divinorum* pseudochromosomes (Chr1-Chr11). The color gradient indicates low (yellow) to high (red) contact frequencies.

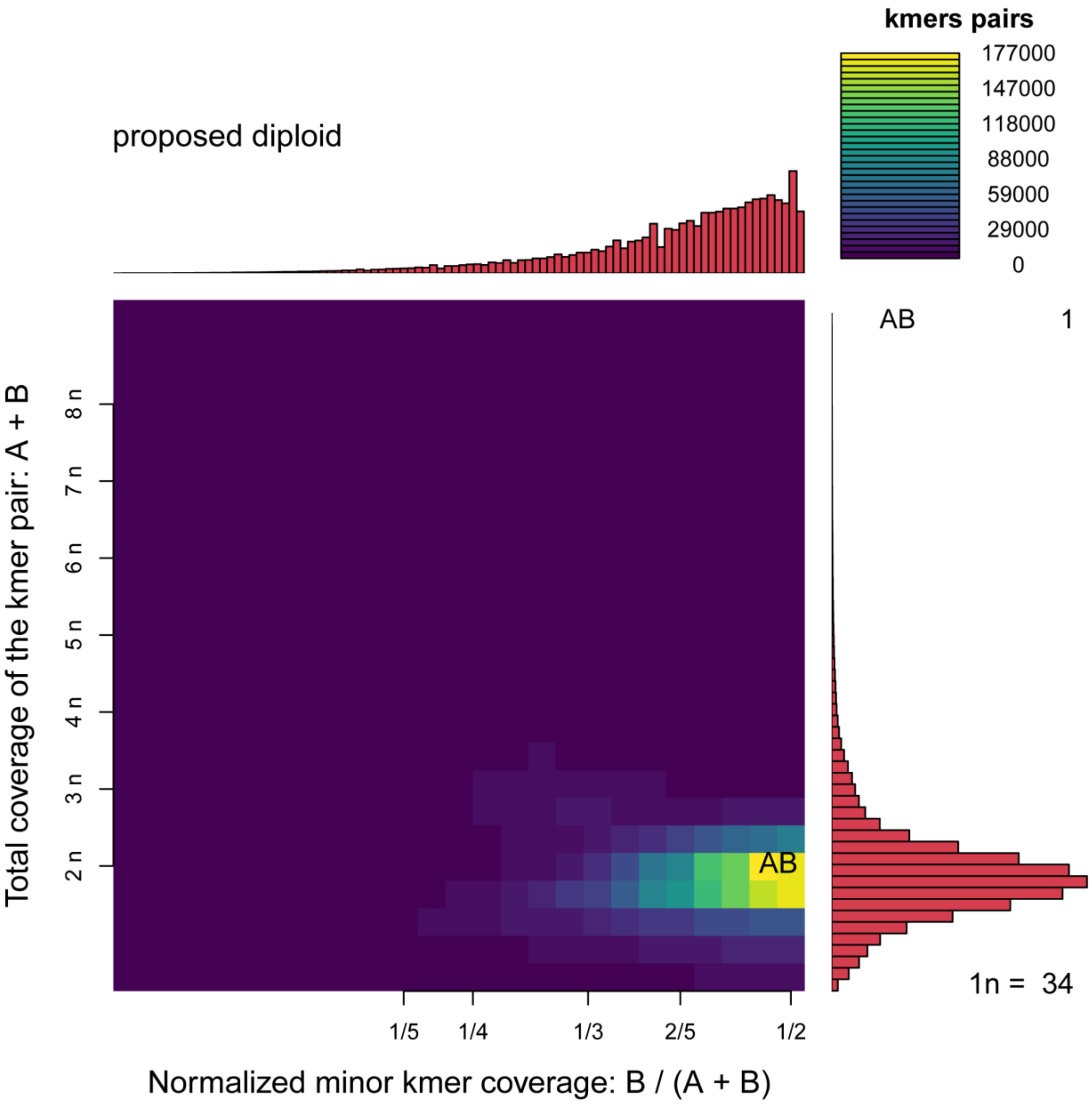

Supplementary Figure 5. Smudgeplot analysis of *S. divinorum*.

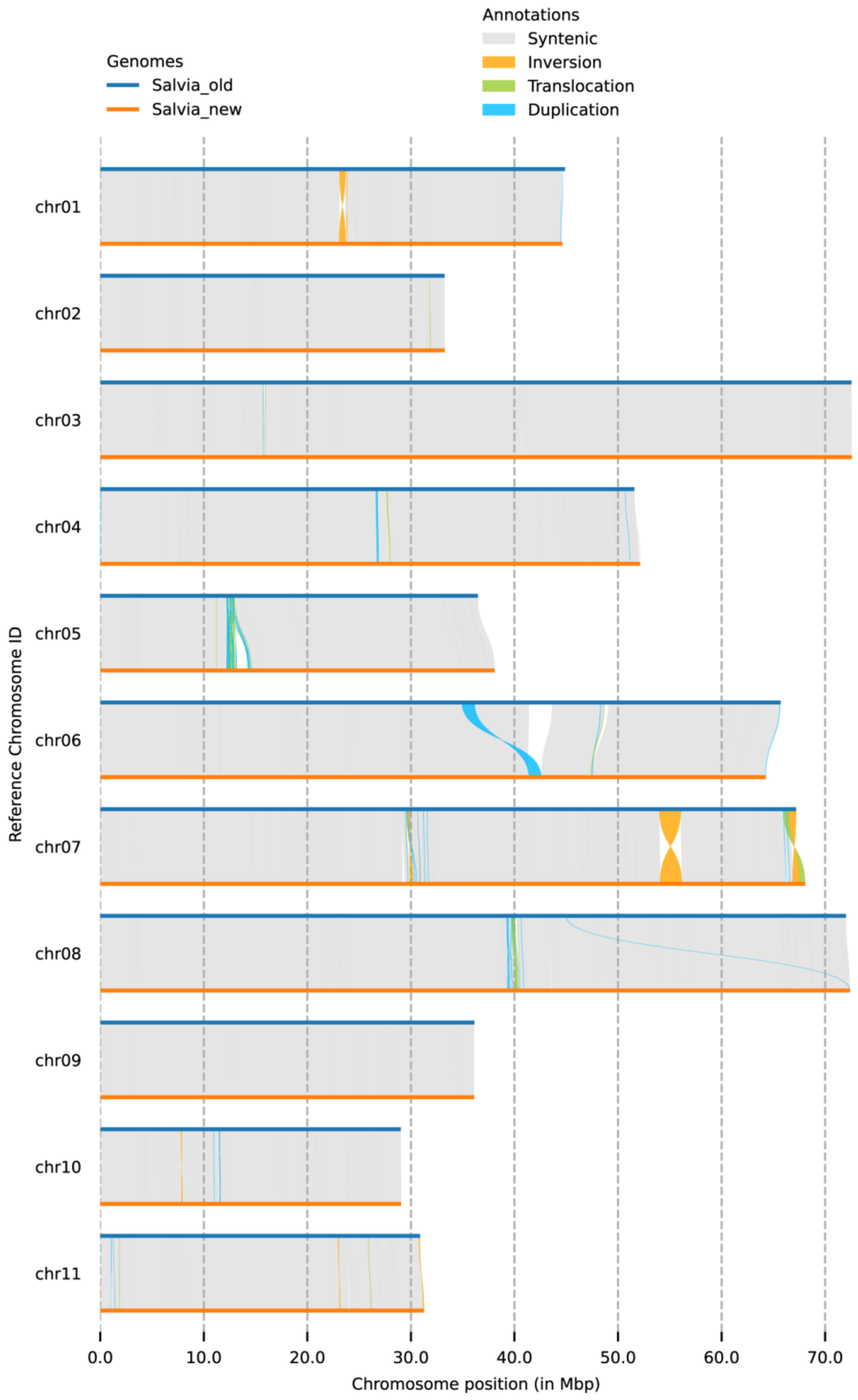

Supplementary Figure 6. Comparison of the current current genome of *S. divinorum* published in this study compared with an earlier version[1] using plotsr. Source data are provided as a Source Data file.

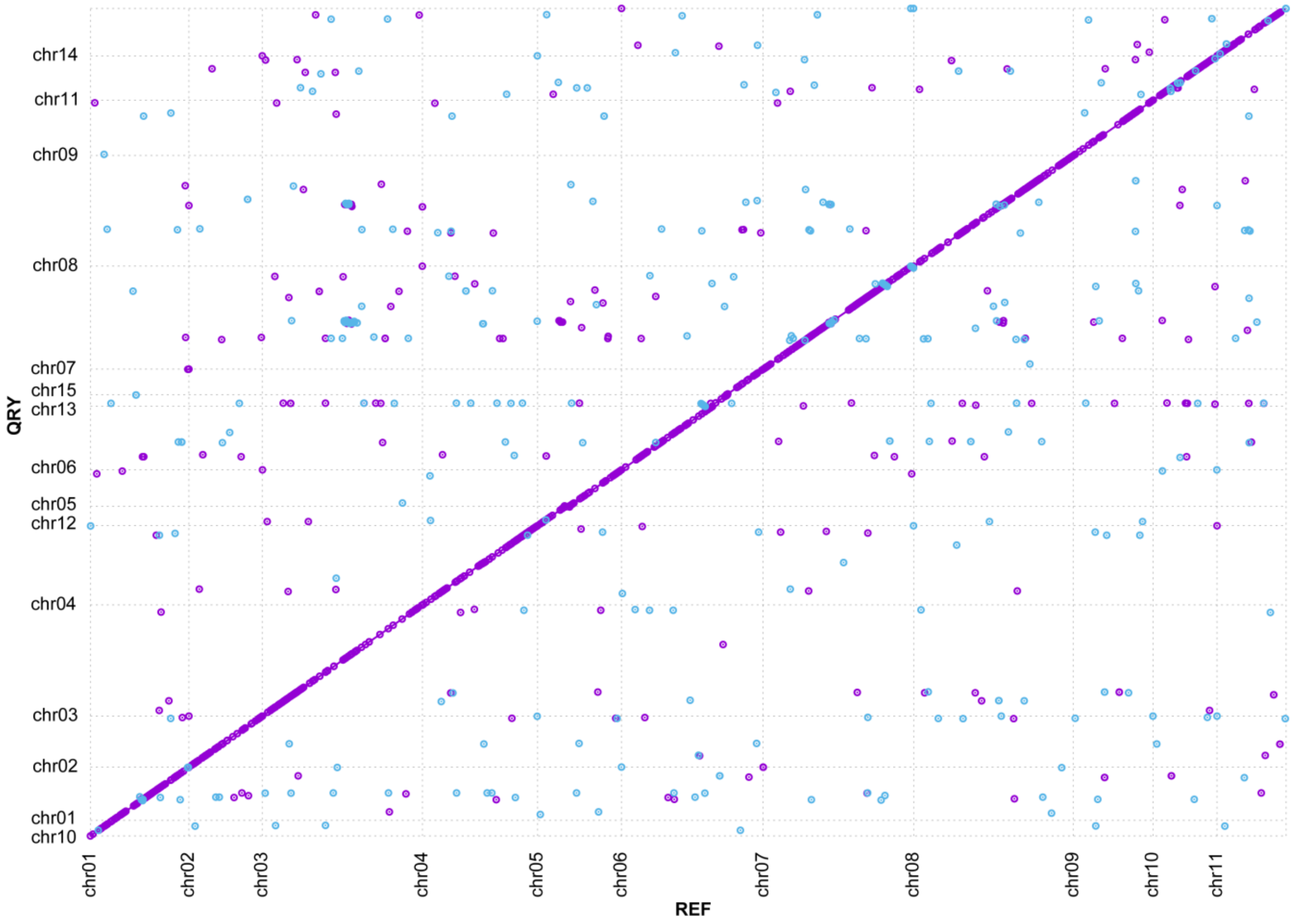

Supplementary Figure 7. Comparison of the current current reference genome of *S. divinorum* published in this study compared with an earlier version[1] using Mummer. The horizontal axis represents the *S. divinorum* reference genome at chromosome level (published in this study). The vertical axis represents the previous version of *S. divinorum* genome (NCBI accession number: GCA_041381175.1)[1].

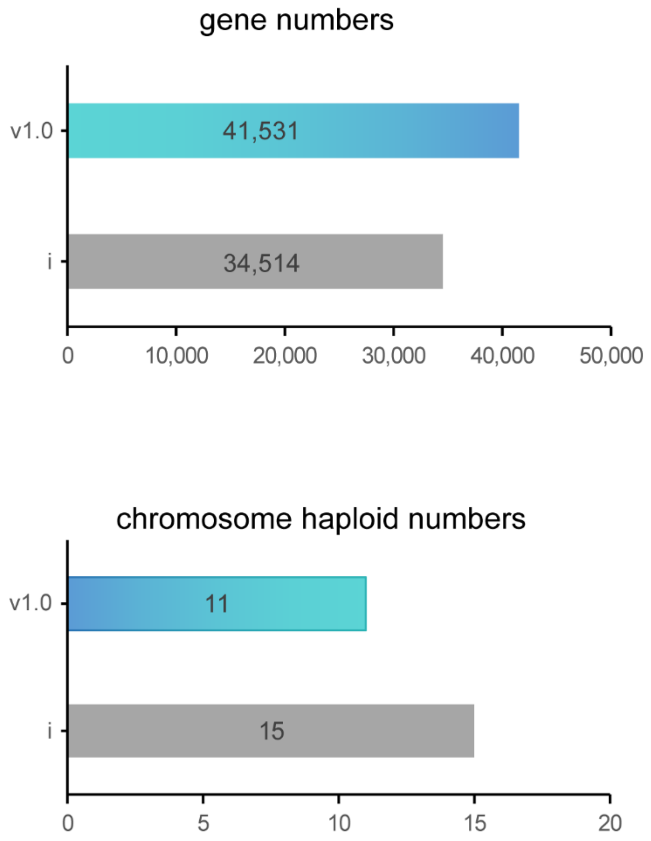

Supplementary Figure 8. Differences between the reference genome of *S. divinorum* published in this study (blue bars) and the previous version (grey bars) [1].

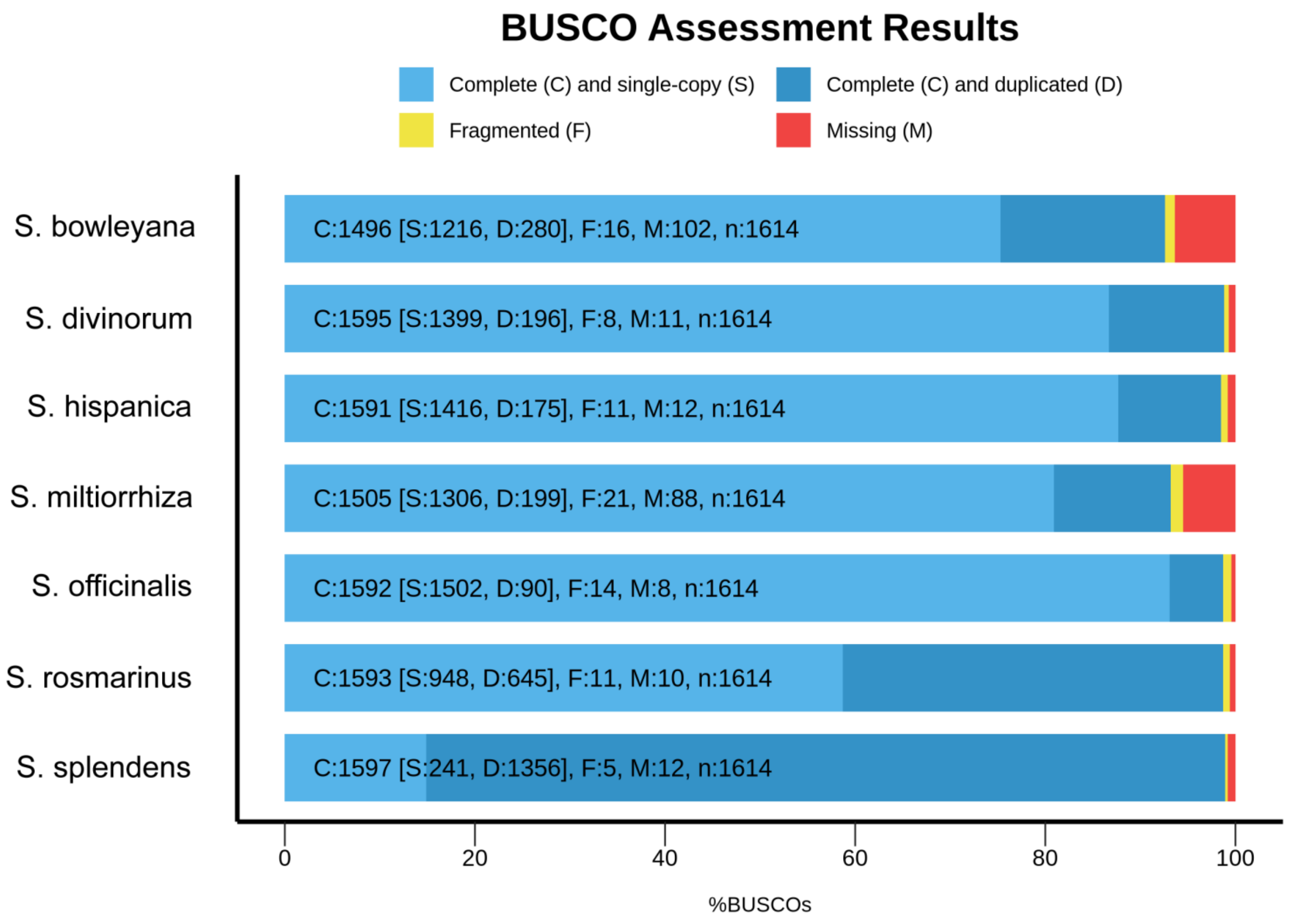

Supplementary Figure 9. BUSCO assessment results among genomes of different *Salvia* species. The completeness of the assembly was assessed by BUSCO (v5.8.0) using the embryophyta_odb10 database. bowleyana: *S. bowleyana*[2]; divinorum: *S. divinorum*[1]; hispanica: *S. hispanica*[3]; miltiorrhiza: *S. miltiorrhiza*[4]; officinalis: *S. officinalis*[5]; rosmarinus: *S. rosmarinus*[6]; splendens: *S. splendens*[7].

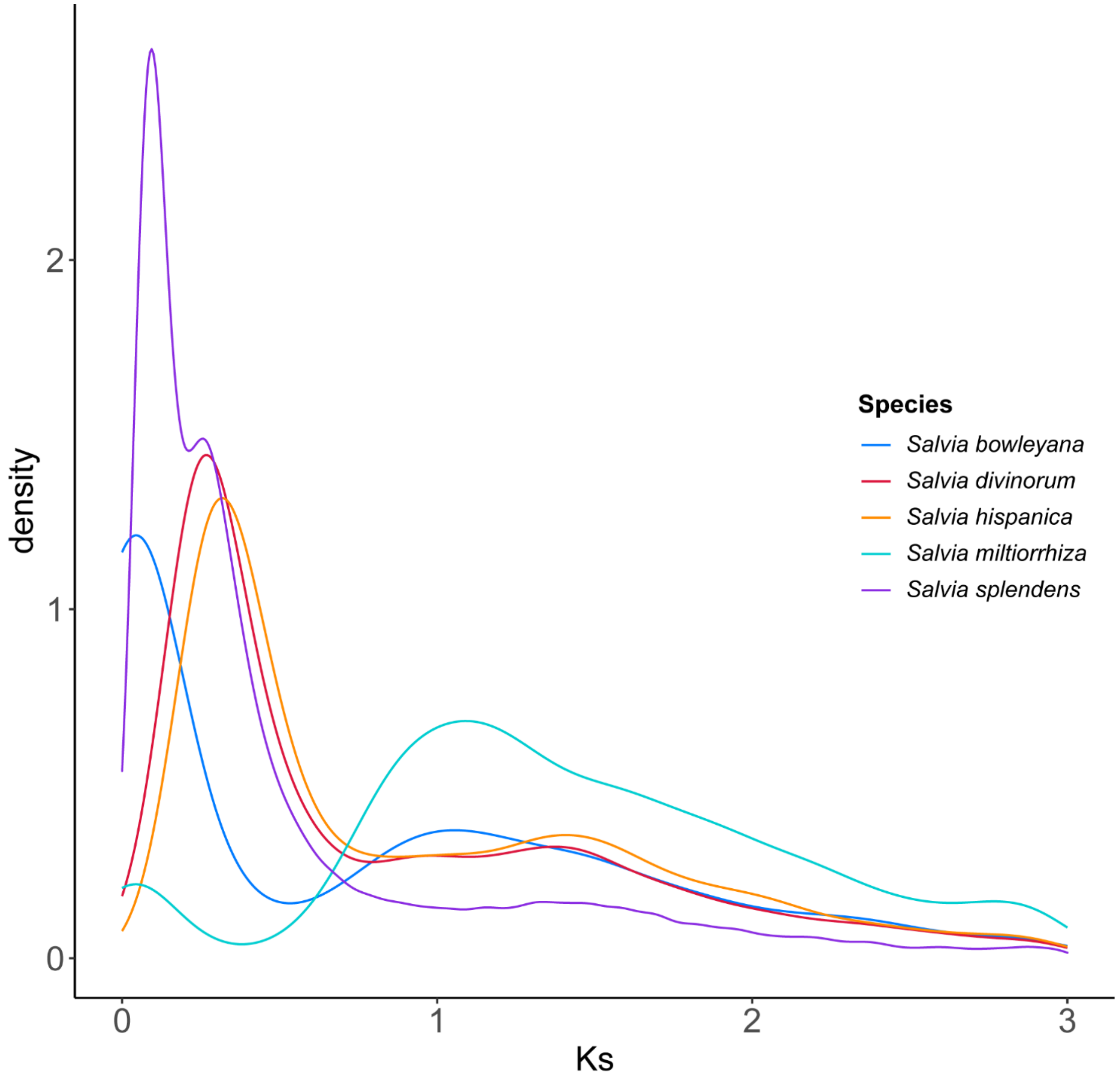

Supplementary Figure 10. Synonymous substitution rate (Ks) distributions for *S. divinorum*, *S. miltiorrhiza*, *S. bowleyana*, *S. splendens*, and *S. hispanica*. Source data are provided as a Source Data file.

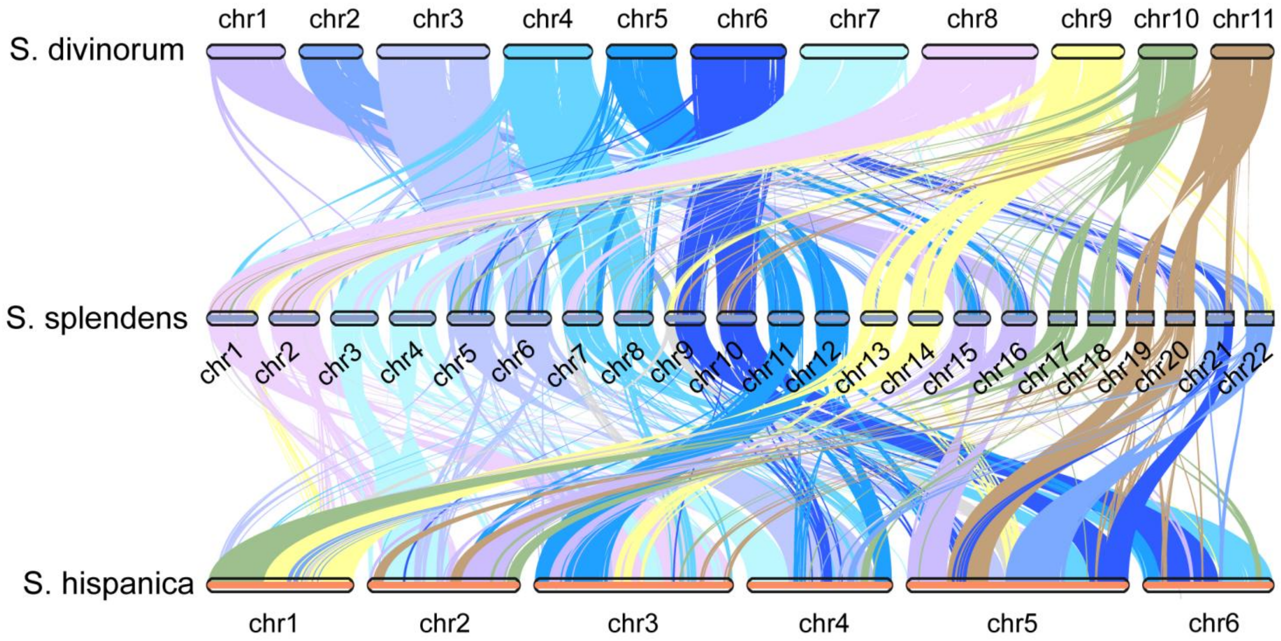

Supplementary Figure 11. Macrosynteny between the three neotropical *Salvia* species *S. divinorum*, *S. splendens*, and *S. hispanica*. Conserved syntenic regions between the chromosomes of *S. divinorum*, *S. hispanica* and *S. splendens* are highlighted with different colors.

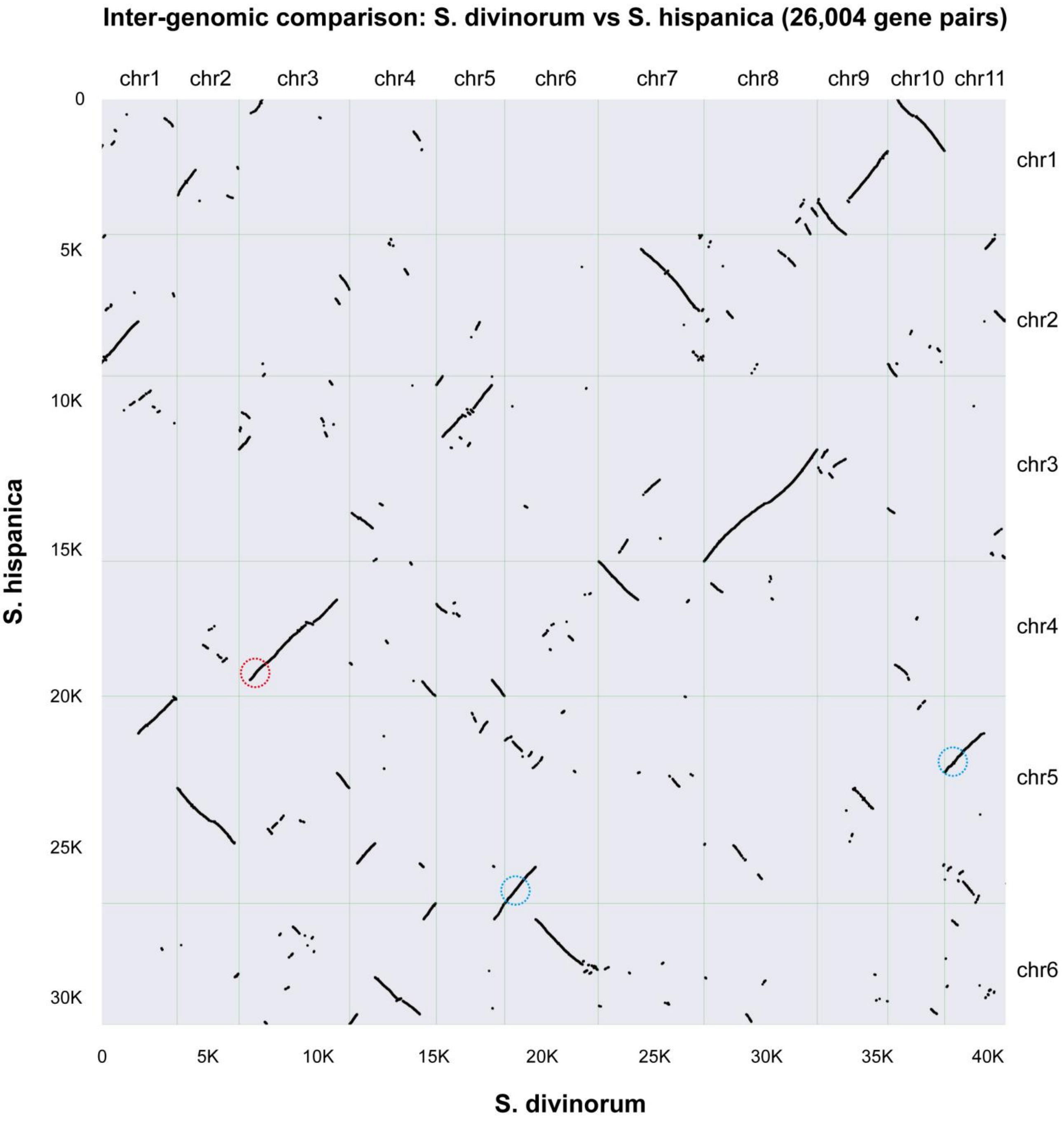

Supplementary Figure 12. Syntenic dot plots between *S. divinorum* and *S. hispanica*. Source data are provided as a Source Data file.

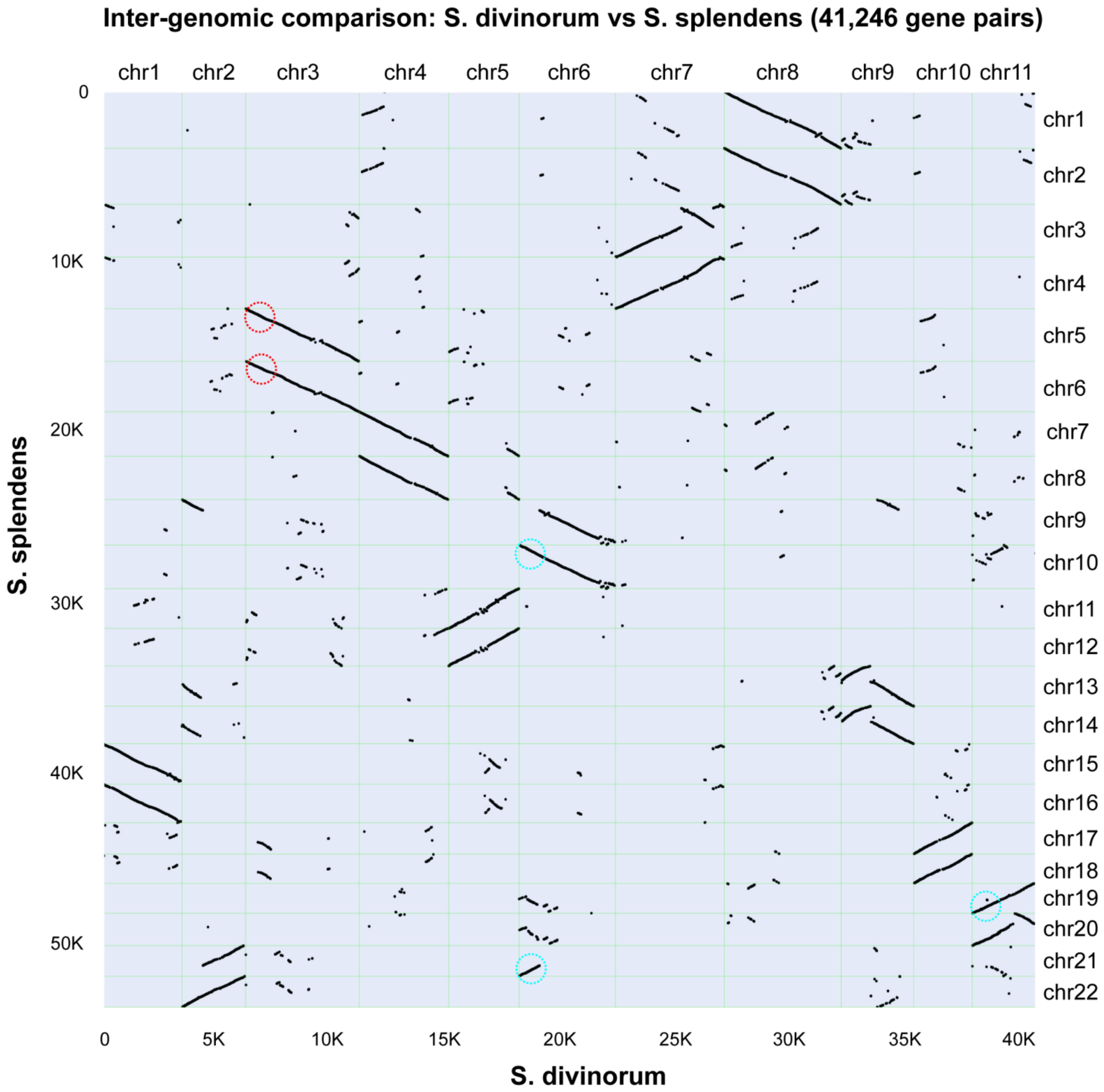

Supplementary Figure 13. Syntenic dot plots between *S. divinorum* and *S. splendens*. Source data are provided as a Source Data file.

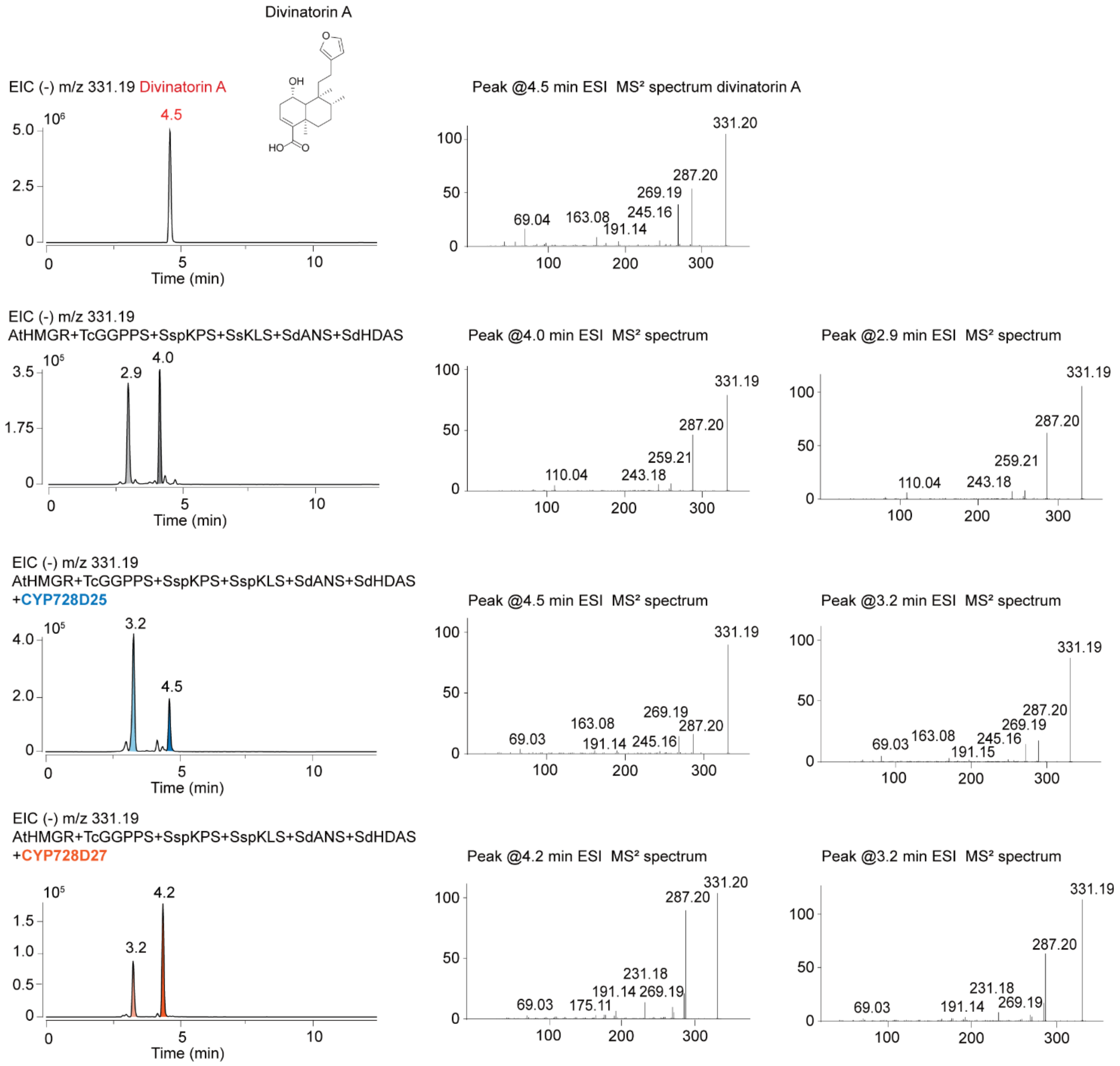

Supplementary Figure 14. LC-MS analysis of divinatorin A standard and its isomers from combined expression of *S. divinorum* cytochrome P450 enzymes CYP728D25 and CYP728D27with upstream enzymes in furanoclerodane metabolism. LC-MS analysis of divinatorin A standard isolated from *Dodonaea viscosa* (selected *m/z* signal of 331.19 in negative mode). MS^2 spectra of peak at 4.5 min. with 45 % high-energy collisional dissociation (HCD). LC-MS analysis of extracts (Extracted Ion Chromatogram at *m/z* 331.19 in negative mode) of infiltrated *N. benthamiana* leaves with *Agrobacterium* strains carrying AtHMGR, TcGGPPS, SspKPS, SspKLS, SdANS, SdHDAS with CYP728D25 (SdDAS). MS^2 spectra of peaks eluting at 3.2 (from 331.19 mother ion) and 4.5 min (from 331.19 mother ion) are shown. LC-MS analysis of extracts (Extracted Ion Chromatogram at *m/z* 331.19 in negative mode) of infiltrated *N. benthamiana* leaves with *Agrobacterium* strains carrying AtHMGR, TcGGPPS, SspKPS, SspKLS, SdANS, SdHDAS with CYP728D27. MS^2 spectra of peaks eluting at 4.2 (from 331.19 mother ion) and 3.2 min (from 331.19 mother ion) are shown.

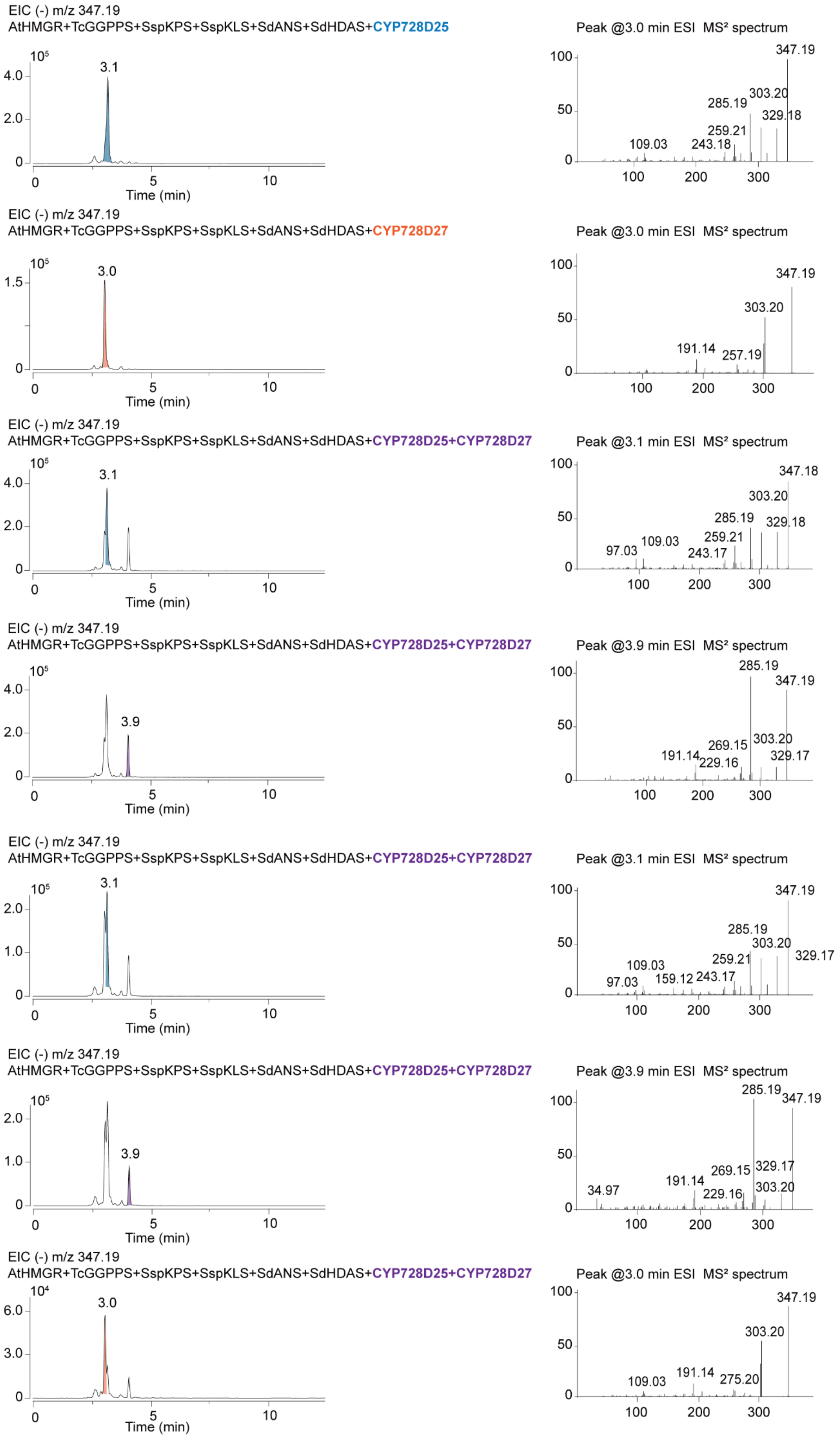
EIC (-) m/z 347.19
AtHMGR+TcGGPPS+SspKPS+SspKLS+SdANS+SdHDAS+CYP728D25
Peak @3.0 min ESI MS² spectrum
3.1
347.19
303.20
285.19
329.18
259.21
109.03
243.18
EIC (-) m/z 347.19
AtHMGR+TcGGPPS+SspKPS+SspKLS+SdANS+SdHDAS+CYP728D27
Peak @3.0 min ESI MS² spectrum
3.0
347.19
303.20
191.14
257.19
EIC (-) m/z 347.19
AtHMGR+TcGGPPS+SspKPS+SspKLS+SdANS+SdHDAS+CYP728D25+CYP728D27
Peak @3.1 min ESI MS² spectrum
3.1
347.18
303.20
285.19
329.18
259.21
109.03
97.03
243.17
EIC (-) m/z 347.19
AtHMGR+TcGGPPS+SspKPS+SspKLS+SdANS+SdHDAS+CYP728D25+CYP728D27
Peak @3.9 min ESI MS² spectrum
3.9
285.19
347.19
269.15
303.20
191.14
229.16
329.17
EIC (-) m/z 347.19
AtHMGR+TcGGPPS+SspKPS+SspKLS+SdANS+SdHDAS+CYP728D25+CYP728D27
Peak @3.1 min ESI MS² spectrum
3.1
347.19
285.19
303.20
329.17
109.03
259.21
97.03
159.12
243.17
EIC (-) m/z 347.19
AtHMGR+TcGGPPS+SspKPS+SspKLS+SdANS+SdHDAS+CYP728D25+CYP728D27
Peak @3.9 min ESI MS² spectrum
3.9
285.19
347.19
329.17
269.15
191.14
34.97
229.16
303.20
EIC (-) m/z 347.19
AtHMGR+TcGGPPS+SspKPS+SspKLS+SdANS+SdHDAS+CYP728D25+CYP728D27
Peak @3.0 min ESI MS² spectrum
3.0
347.19
303.20
191.14
109.03
275.20
Time (min)

Supplementary Figure 15. LC-MS analysis of double hydroxylated products of hardwickiic acid by expression of *S. divinorum* cytochrome P450 enzymes CYP728D25 and CYP728D27with upstream enzymes in furanoclerodane metabolism. LC-MS analysis of extracts (Extracted Ion Chromatogram at *m/z* 347.19 in negative mode) of infiltrated *N. benthamiana* leaves with *Agrobacterium* strains carrying AtHMGR, TcGGPPS, SspKPS, SspKLS, SdANS, SdHDAS with CYP728D25 (SdDAS). MS^2 spectrum of peak eluting at 3.1 (from 347.19 mother ion) is shown. LC-MS analysis of extracts (Extracted Ion Chromatogram at *m/z* 347.19 in negative mode) of infiltrated *N. benthamiana* leaves with *Agrobacterium* strains carrying AtHMGR, TcGGPPS, SspKPS, SspKLS, SdANS, SdHDAS with CYP728D27. MS^2 spectrum of peak eluting at 3.0 (from 347.19 mother ion) is shown. LC-MS analysis of extracts (Extracted Ion Chromatogram at *m/z* 347.19 in negative mode) of infiltrated *N. benthamiana* leaves with *Agrobacterium* strains carrying AtHMGR, TcGGPPS, SspKPS, SspKLS, SdANS, SdHDAS, SdDAS (CYP728D25) with CYP728D27. MS^2 spectrA of peaks eluting at 3.0 (from 347.19 mother ion), 3.1 (from 347.19 mother ion), and 3.9 min (from 347.19 mother ion) are shown.

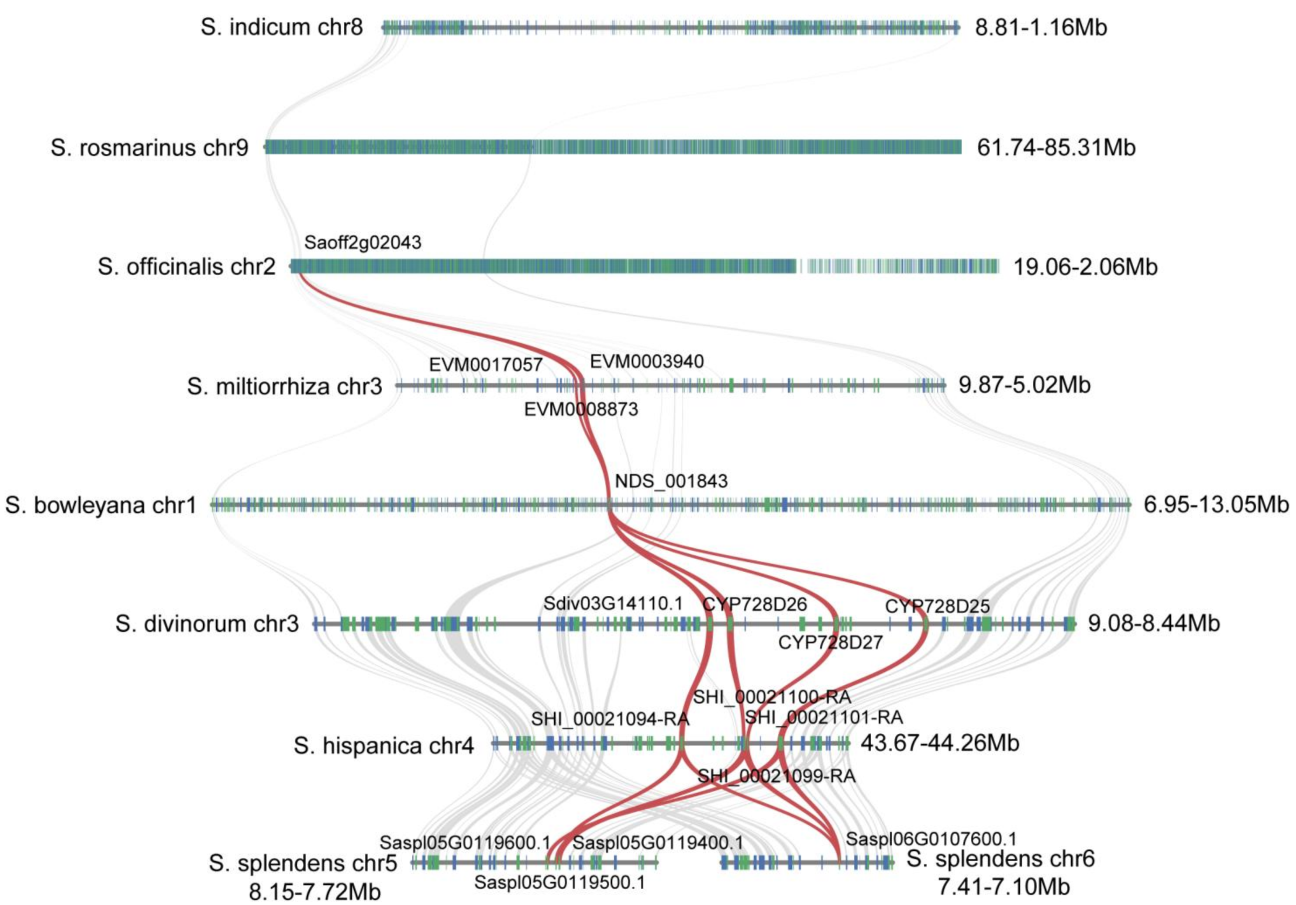

Supplementary Figure 16. Extended syntenic analysis of the genes encoding CYP728D enzymes in the neotropical sages *S. divinorum*, *S. splendens*, *S. hispanica*, Asian sages *S. miltiorrhiza*, and *S. bowleyana*, and European sages *S. rosmarinus*, and *S. officinalis*. Red ribbons highlight the syntenic relationships between CYP728D genes.

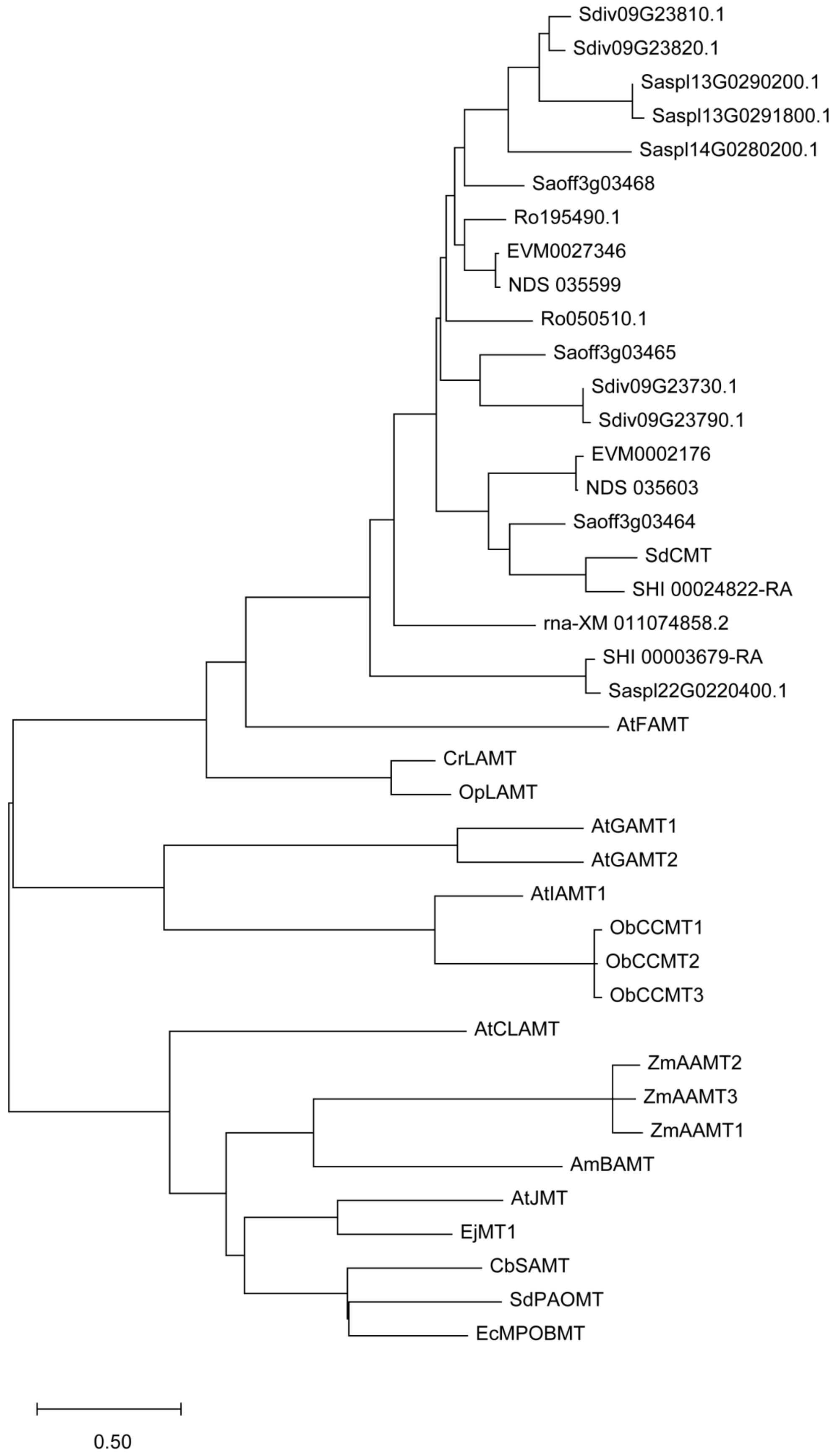

Supplementary Figure 17. Phylogenetic tree of functionally characterized SABATH family methyl transferases (mainly carboxy methyl transferases) including the methyl transferases syntenic to SdCMT in other *Salvia* plant species. Analytical description of each enzyme (plant origin, activity, etc) are provided in Supplementary Table 8. Source data are provided as a Source Data file.

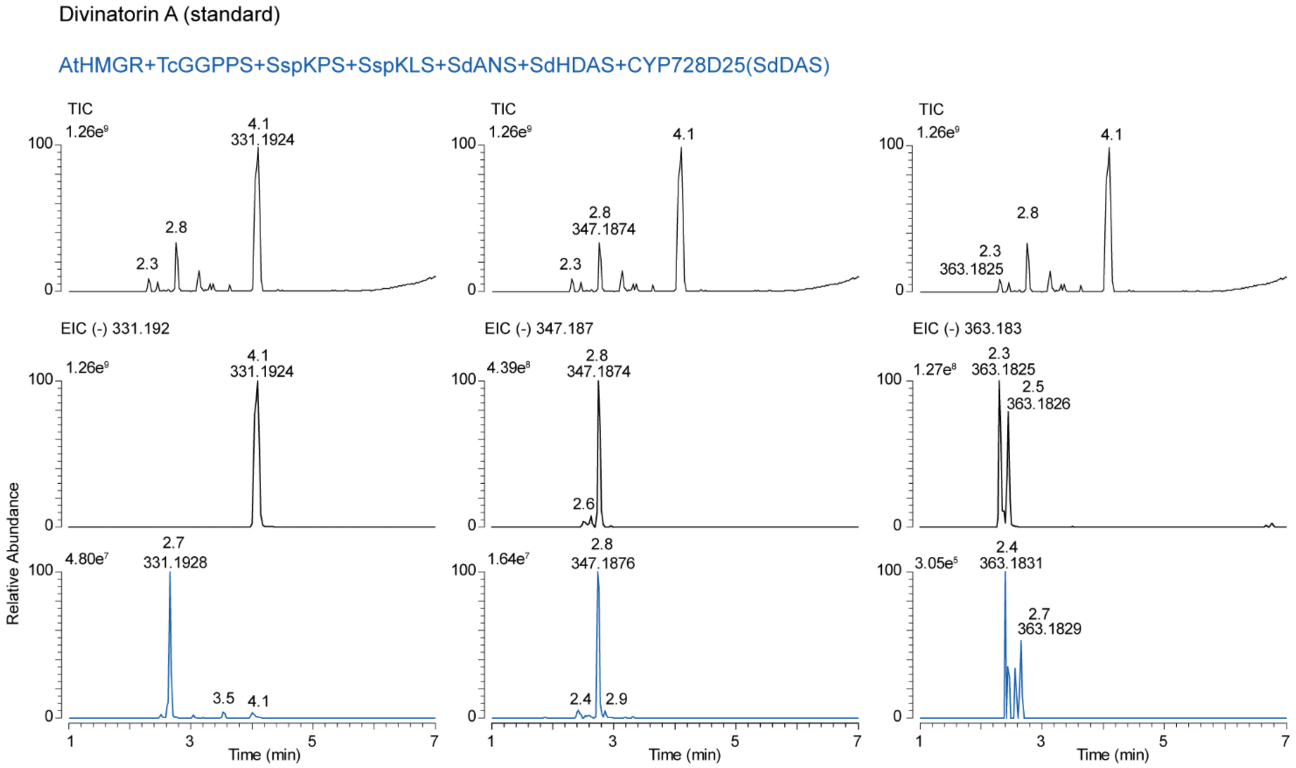

Supplementary Figure 18. Observed chemical oxidations on divinatorin A standard by LC-MS during sample preparation and analysis. First row: Total Ion Chromatogram (TIC) of isolated divinatorin A standard. Second row: Extracted Ion Chromatograms at *m/z* 331.192, 347.187, and 363.183 of isolated divinatorin A sample. Third row: Extracted Ion Chromatograms at *m/z* 331.192, 347.187, and 363.183 from assays of CYP728D25 in *N. benthamiana*.

Supplementary Table 1. Flow Cytometry fluorescene intensity prediction of *S. divinorum* genome size.

**Species**	**Sample 1**	**Sample 2**	**Sample 3**	**Fluorescence intensity mean**
Solanum lycopersicum	2667.31	2698.66	2710.82	2692.26
S. divinorum	1387.8	1401.57	1389.31	1392.89

Supplementary Table 2. k-mer analysis results for *S. divinorum* genome size estimation results by GCE (genomic charactor estimator) and GenomeScope.

GCE results							
**k-mer**	**Raw peak**	**Effective k-mer species**	**Effective mer individuals**	**Coverage depth**	**Genome size**	**a[1]**	**b[1]**
17	35	240514094	19628830550	35.4704	553,000,000	0.81033	0.34306
19	34	278383853	19266442377	34.6719	556,000,000	0.86875	0.42436
Genomescope results: k-mer 17, 550 Mb; k-mer 19, 551 Mb; k-mer 21, 551 Mb; k-mer 23, 552 Mb.							
Genomescope2 results: k-mer 17, 273 Mb; k-mer 19, 551 Mb; k-mer 21, 551 Mb; k-mer 23, 551 Mb.							

Note:
a[1]：The proportion of unique k-mers among all k-mer types in the genome.
b[1]：The proportion of the total count of k-mers that appear only once in the genome to the total count of all k-mers.

Supplementary Table 3. Parameters of genome assembly before, removing redundancy by purde_dups and after Hi-C.

**Parameters of original genome assembly**				
	Contig			
	Length (bp)	Number		
N10	72,379,191	1		
N20	66,849,701	2		
N30	44,623,150	3		
N40	40,717,687	5		
N50	36,113,457	6		
N60	31,844,902	8		
N70	29,056,797	10		
N80	25,036,219	12		
N90	21,691,201	14		
Total length	574,586,498			
contig number	410			
GC rate	37.29%			
**Assembly treated by purge_dups**				
	Contig			
	Length (bp)	Number		
N10	72,379,191	1		
N20	66,849,701	2		
N30	44,623,150	3		
N40	42,581,071	4		
N50	36,113,457	6		
N60	33,268,200	7		
N70	31,172,984	9		
N80	27,894,600	11		
N90	24,148,344	13		
Total length	558,956,549	---		
contig number	215	---		
GC rate	37.34%	---		
**Assembly improvement combined with HiC data using 3D-DNA**				
	Contig	Super-scaffold		
	Length (bp)	Number	Length (bp)	Number
max_length	72,379,191		72,591,418	
N50	36,113,457	6	52,135,465	5
N60	33,268,200	7	44,623,150	6
N70	31,172,984	9	38,083,006	7
N80	36,113,457	8	27,894,600	11
N90	31,255,382	10	24,148,344	13
Total	558,956,549	----	558,961,049	----
Anchored rate (%)	96.94%			

Supplementary Table 4. BUSCO analysis of the *S. divinorum* genome assembly after removing redundancy by purde_dups.

**Gene mode**		
Type	Number	Rate（%）
Complete BUSCOs (C)	1,595	98.8
Complete and single-copy BUSCOs (S)	1,399	86.7
Complete and duplicated BUSCOs (D)	196	12.1
Fragmented BUSCOs (F)	8	0.5
Missing BUSCOs (M)	11	0.7
Total BUSCOs groups searched	1,614	
**Protein Mode**		
Type	Number	Rate（%）
Complete BUSCOs (C)	1,601	99.2
Complete and single-copy BUSCOs (S)	1,408	87.2
Complete and duplicated BUSCOs (D)	193	12.0
Fragmented BUSCOs (F)	3	0.2
Missing BUSCOs (M)	10	0.6
Total BUSCOs groups searched	1,614	
# BUSCO version is: 5.8.2		
# The lineage dataset is: embryophyta_odb10 (Creation date: 2024-01-08, number of genomes: 50, number of BUSCOs: 1614)		
# Summarized benchmarking in BUSCO notation for file Salvia_divinorum.v2.representative.pep.fa		
Dependencies and versions:		
hmmsearch: 3.3		
python: sys.version_info(major=3, minor=10, micro=13, releaselevel='final', serial=0)		
busco: 5.8.2		

Supplementary Table 5. Total length grouped on pseudochromosomes of *S. divinorum*.

Chromosome	Total length (bp)	N region (bp)	Effective length (bp)
chr01	44,623,150	0	44,623,150
chr02	33,268,200	0	33,268,200
chr03	72,591,418	1,000	72,590,418
chr04	52,135,465	1,000	52,134,465
chr05	38,083,006	500	38,082,506
chr06	64,272,772	500	64,272,272
chr07	68,073,698	500	68,073,198
chr08	72,403,737	500	72,403,237
chr09	36,113,457	0	36,113,457
chr10	29,056,797	0	29,056,797
chr11	31,255,382	500	31,254,882
Total	541,877,082	4,500	541,872,582

Supplementary Table 6. Repetitive DNA composition in the *S. divinorum* genome.

Type	Length (bp)	% in genome
DNA transposable elements	17747811	3.175143
LINE	9524511	1.703967
SINE	20547	0.003676
LTR	312705391	55.94404
Other	3261	0.000583
Unknown	51192811	9.158565
Total	405569226	72.55769

Supplementary Table 7. Non-coding RNA composition in the *S. divinorum* genome.

Type	Subtype	Number of copies identified across the whole genome
**miRNA**		94
**tRNA**		1880
**rRNA**	rRNA	8862
	18S	963
	28S	2490
	5.8S	610
	5S	4799
**snRNA**	snRNA	653
	CD-box	159
	HACA-box	30
	splicing	464

Supplementary Table 8. List of plant carboxy methyltransferases (SABATH) used for phylogenetic trees in Figure 9 and Supplementary Figure 16.

**Enzyme name and abbreviation**	**Plant species**	**Structure PDB code/NCBI accesion**
Salicylic acid methyltransferase (SAMT) [8,9]	*Clarkia breweri*	1M6E/Q9SPV4.1
Benzoic acid methyltransferase (BAMT) [10]	*Antirrhinum majus*	Q9FYZ9.1
Jasmonic acid methyltransferase (JMT) [11]	*Arabidopsis thaliana*	NP_173394.1
Indole-3-acetic acid methyltransferase (IAMT) [9,12,13]	*Arabidopsis thaliana*	3B5I
Farnesoic acid methyltransferase (FAMT) [14]	*Arabidopsis thaliana*	Q9FYC4.1
Gibberellic acid methyltransferase (GAMT1/2) [15]	*Arabidopsis thaliana*	F4JUY5.1
Loganic acid methyltransferase (LAMT) [16-18]	*Catharanthus roseus*	Q5XF78.1<br>6C8S 6C8R
Anthranilic acid methyltransferase (AAMT1/2/3) [19]	*Zea mays* (maize)	ADI87450.1<br>ADI87451.1<br>ADI87452.1
Cinnamate/*p*-coumarate methyltransferase (CCMT1/2/3) [20]	*Ocimum basilicum*	ABV91100.1<br>ABV91101.1<br>ABV91102.1
Perillic acid (PA) methyltransferase (SdPAOMT) [21]	*S. dorisiana*	QCF60516.1
Carlactonoic acid methyltransferase [22]	*Arabidopsis thaliana*	O23234.2
4-(1-methyl-2-pyrrolidinyl)-3-oxobutanic acid (MPOB) methyltransferase [23]	*Erythroxylum coca*	WAU46224.1
CcDXMT [24]	*Coffea canephora*	2EFJ/ABD90686
CcXMT [24]	*Coffea canephora*	2EG5/ABD90685
OpLAMT [25]	*Ophiorrhiza pumila*	QWX38535
EjMBMT [26]	*Eriobotyra japonica*	BAV54103

Supplementary Table 9. List of primers used in this study.

Gene name or Gene ID	Primer name	Sequence (5' to 3')
AtHMGR	pTRBO-F	AAGTTCTGTTTCAGGGCCCGATGGATCTCCGTCGGAGGCC
	pTRBO-R	ATGGTCTAGAAAGCTTTATCATGTTGTTGTTGTTGTCGTTGTCG
TcGGPPSΔ18	pEAQ-F	ttctgcccaaattcgcgaccggtATGGCTTCCTATCAAGAATGC
	pEAQ-R	aaccagagttaaaggcctcgagTCAGTTTTGCCTGAATGCA
SsKPSΔ72	pEAQ-F	ttctgcccaaattcgcgaccggtATGTCAACAATTTTGGAGGGACAAAC
	pEAQ-R	aaccagagttaaaggcctcgagCTAGACAATTTTTTCAAACA
SsKLSΔ50	pEAQ-F	ttctgcccaaattcgcgaccggtATGTTGGGGGAATTAAAGGACAAG
	pEAQ-R	aaccagagttaaaggcctcgagTTAACTAGGAAGTGTAAGAGG
SdANS	pEAQ-F	ttctgcccaaattcgcgaccggtATGGATTCCTTCCCTTTCCTC
	pEAQ-R	aaccagagttaaaggcctcgagTCATAACTTATATGGGATGC
CYP728D26	pEAQ-F	ttctgcccaaattcgcgaccggtATGGAGTCGACGATTATGTTG
	pEAQ-R	aaccagagttaaaggcctcgagTTATATTATATTTGTCTGCATTG
CYP728D25	pEAQ-F	ttctgcccaaattcgcgaccggtATGGAGTCGACGATTATGTTG
	pEAQ-R	aaccagagttaaaggcctcgagTTAGAAAGGGTTGGTGATGTG
SdCMT	pEAQ-F	ttctgcccaaattcgcgaccggtATGCATCATATCAATCTCAC
	pEAQ-R	AACCAGAGTTAAAGGCCTCGAGTTATTTGCGCTTCAACACAACAAAG
Sdiv03G06170	pEAQ-F	TTCTGCCCAAATTCGCGACCGGTATGGTGCTAGATGAAGAAGTG
	pEAQ-R	AACCAGAGTTAAAGGCCTCGAGTTATCATGGAAAGGCCTCAATAAC
Sdiv03G06180	pEAQ-F	TTCTGCCCAAATTCGCGACCGGTATGTCAAAAATCACATTAAAAAAG
	pEAQ-R	AACCAGAGTTAAAGGCCTCGAGTTTTAGGGATATGCCTCAATGAC
CYP728D27	pEAQ F	ttctgcccaaattcgcgaccggtATGGAGTCGACGATTATGTTG
	pEAQ R	aaccagagttaaaggcctcgagTCAGAAAGGGTTGGTGATGTGAATATG

Supplementary references

1 Ford, S. A. *et al.* A chromosome level reference genome of Diviner's sage (*Salvia divinorum*) provides insight into salvinorin A biosynthesis. *BMC Plant Biology* **24**, 914 (2024).

2 Zheng, X. *et al.* Insights into salvianolic acid B biosynthesis from chromosome-scale assembly of the *Salvia bowleyana* genome. *Journal of Integrative Plant Biology* **63**, 1309-1323 (2021).

3 Wang, L. *et al.* A chromosome-level genome assembly of chia provides insights into high omega-3 content and coat color variation of its seeds. *Plant Communications* **3**, 100326 (2022).

4 Pan, X. *et al.* Chromosome-level genome assembly of *Salvia miltiorrhiza* with orange roots uncovers the role of Sm2OGD3 in catalyzing 15,16-dehydrogenation of tanshinones. *Horticulture Research* **10** (2023).

5 Li, C.-Y. *et al.* The sage genome provides insight into the evolutionary dynamics of diterpene biosynthesis gene cluster in plants. *Cell Reports* **40**, 111236 (2022).

6 Han, D. *et al.* The chromosome-scale assembly of the *Salvia rosmarinus* genome provides insight into carnosic acid biosynthesis. *Plant J* **113**, 819-832 (2023).

7 Jia, K.-H. *et al.* Chromosome-scale assembly and evolution of the tetraploid *Salvia splendens* (Lamiaceae) genome. *Horticulture Research* **8**, 177 (2021).

8 Ross, J. R. *et al.* *S*-adenosyl-L-methionine:salicylic acid carboxyl methyltransferase, an enzyme involved in floral scent production and plant defense, represents a new class of plant methyltransferases. *Arch Biochem Biophys* **367**, 9-16 (1999).

9 Zubieta, C. *et al.* Structural basis for substrate recognition in the salicylic acid carboxyl methyltransferase family. *Plant Cell* **15**, 1704-1716 (2003).

10 Murfitt, L. M. *et al.* Purification and characterization of *S*-adenosyl-L-methionine:benzoic acid carboxyl methyltransferase, the enzyme responsible for biosynthesis of the volatile ester methyl benzoate in flowers of *Antirrhinum majus*. *Arch Biochem Biophys* **382**, 145-151 (2000).

11 Seo, H. S. *et al.* Jasmonic acid carboxyl methyltransferase: a key enzyme for jasmonate-regulated plant responses. *Proc Natl Acad Sci U S A* **98**, 4788-4793 (2001).

12 Qin, G. *et al.* An indole-3-acetic acid carboxyl methyltransferase regulates Arabidopsis leaf development. *Plant Cell* **17**, 2693-2704 (2005).

13 Zhao, N. *et al.* Structural, biochemical, and phylogenetic analyses suggest that indole-3-acetic acid methyltransferase is an evolutionarily ancient member of the SABATH family. *Plant Physiol* **146**, 455-467 (2008).

14 Yang, Y. *et al.* An *Arabidopsis thaliana* methyltransferase capable of methylating farnesoic acid. *Arch Biochem Biophys* **448**, 123-132 (2006).

15 Varbanova, M. *et al.* Methylation of gibberellins by Arabidopsis GAMT1 and GAMT2. *Plant Cell* **19**, 32-45 (2007).

16 Madyastha, K. M. *et al*. S-Adenosyl-L-methionine: Loganic acid methyltransferase. A carboxyl-alkylating enzyme from *Vinca rosea*. *J Biol Chem* **248**, 2497-2501 (1973).

17 Murata, J. *et al*. The leaf epidermome of *Catharanthus roseus* reveals its biochemical specialization. *Plant Cell* **20**, 524-542 (2008).

18 Petronikolou, N. *et al*. Loganic acid methyltransferase: Insights into the specificity of methylation on an iridoid glycoside. *Chembiochem* **19**, 784-788 (2018).

19 Köllner, T. G. *et al.* Herbivore-induced SABATH methyltransferases of maize that methylate anthranilic acid using *s*-adenosyl-L-methionine. *Plant Physiol* **153**, 1795-1807 (2010).

20 Kapteyn, J. *et al.* Evolution of Cinnamate/*p*-coumarate carboxyl methyltransferases and their role in the biosynthesis of methylcinnamate. *Plant Cell* **19**, 3212-3229 (2007).

21 Jongedijk, E. *et al.* Novel routes towards bioplastics from plants: elucidation of the methylperillate biosynthesis pathway from *Salvia dorisiana* trichomes. *J Exp Bot* **71**, 3052-3065 (2020).

22 Mashiguchi, K. *et al.* A carlactonoic acid methyltransferase that contributes to the inhibition of shoot branching in Arabidopsis. *Proc Natl Acad Sci U S A* **119**, e2111565119 (2022).

23 Chavez, B. G. *et al.* Elucidation of tropane alkaloid biosynthesis in *Erythroxylum coca* using a microbial pathway discovery platform. *Proc Natl Acad Sci U S A* **119**, e2215372119 (2022).

24 McCarthy, A. A. *et al.* Cloning, expression, crystallization and preliminary X-ray analysis of the XMT and DXMT N-methyltransferases from *Coffea canephora* (robusta). *Structural Biology and Crystallization Communications* **63**, 304-307 (2007).

25 Yang, M. *et al.* Divergent camptothecin biosynthetic pathway in *Ophiorrhiza pumila*. *BMC Biol* **19**, 122 (2021).

26 Koeduka, T. *et al.* Benzenoid biosynthesis in the flowers of *Eriobotrya japonica*: Molecular cloning and functional characterization of *p*-methoxybenzoic acid carboxyl methyltransferase. *Planta* **244**, 725-736 (2016).
