# Supplementary material for: Comparative genome analysis provides a foundation for defining salvinorin A biosynthesis in Salvia divinorum
Source: Nat Commun. 2026 Mar 23;17:3414. doi: 10.1038/s41467-026-70885-3 (PMC13069086; doi:10.1038/s41467-026-70885-3)
Supplement: Supplementary file 3 — Reporting Summary [file 41467_2026_70885_MOESM3_ESM.pdf]

nature portfolio

Corresponding author(s): Cathie Martin, Evangelos Tatsis

Last updated by author(s): 2026/03/04

Reporting Summary

Nature Portfolio wishes to improve the reproducibility of the work that we publish. This form provides structure for consistency and transparency in reporting. For further information on Nature Portfolio policies, see our Editorial Policies and the Editorial Policy Checklist.

Statistics

For all statistical analyses, confirm that the following items are present in the figure legend, table legend, main text, or Methods section.

n/a	Confirmed	
☐	☒	The exact sample size (*n*) for each experimental group/condition, given as a discrete number and unit of measurement
☐	☒	A statement on whether measurements were taken from distinct samples or whether the same sample was measured repeatedly
☐	☒	The statistical test(s) used AND whether they are one- or two-sided<br>*Only common tests should be described solely by name; describe more complex techniques in the Methods section.*
☒	☐	A description of all covariates tested
☒	☐	A description of any assumptions or corrections, such as tests of normality and adjustment for multiple comparisons
☐	☒	A full description of the statistical parameters including central tendency (e.g. means) or other basic estimates (e.g. regression coefficient) AND variation (e.g. standard deviation) or associated estimates of uncertainty (e.g. confidence intervals)
☐	☒	For null hypothesis testing, the test statistic (e.g. *F*, *t*, *r*) with confidence intervals, effect sizes, degrees of freedom and *P* value noted<br>*Give P values as exact values whenever suitable.*
☒	☐	For Bayesian analysis, information on the choice of priors and Markov chain Monte Carlo settings
☒	☐	For hierarchical and complex designs, identification of the appropriate level for tests and full reporting of outcomes
☒	☐	Estimates of effect sizes (e.g. Cohen's *d*, Pearson's *r*), indicating how they were calculated

Our web collection on statistics for biologists contains articles on many of the points above.

Software and code

Policy information about availability of computer code

Data collection	Data collection: LC-MS data were collected and analyzed by Q-Exactive plus (Xcalibur4.4), Waters Xevo G2-XS QTOF (MassLynx V4.2), and SCIEX Zeno TOF 7600 (MSDIAL v5.1.230912).
Data analysis	The sanger sequencing results of amplified genes were analyzed in DNAMAN v6.0.3.40 and Snapgene v4.3.6. The protein sequences were aligned by ClustalW in MEGA11. The phylogenetic trees were constructed in MEGA 11 and OrthoFinder v2.5.4 and visualized with FigTree v1.4.4. Chemical structures were generated in ChemDraw v22.2.0. The assembly of the long reads was carried out using Hifiasm v0.19.9. The data from HI-C sequencing were anchored by juicer v1.5.6 -3Ddna v20190319. The repetitive genomic sequences were identified using RepBase v23.06, RepeatMasker v4.1.6, ltr_finder v1.0.6, and RepeatModeler 2.0.5. Function annotation was based on the SwissProt, TrEMBL, NR, InterProscan v5.71-102.0, SUPERFAMILY, NCBIFAM, PRINTS, Pfam, SMART, ProSiteProfiles, emapperdb v5.0.2 and ProSitePatterns database. The prediction of non-coding RNAs was achieved using tRNAscan-SE v2.0 and Rfam v14.10, BLASTN and INFERNAL v1.1.5. Genomic features were presented by TBtools v2.138. Syntenic analysis was performed by MCscan (Python version). LC-MS data were analyzed further with MSDIal 5.1.230912. Adobe Illustrator 2025 was used to create the figures.

For manuscripts utilizing custom algorithms or software that are central to the research but not yet described in published literature, software must be made available to editors and reviewers. We strongly encourage code deposition in a community repository (e.g. GitHub). See the Nature Portfolio guidelines for submitting code & software for further information.

Data

Policy information about availability of data

All manuscripts must include a data availability statement. This statement should provide the following information, where applicable:

- Accession codes, unique identifiers, or web links for publicly available datasets
- A description of any restrictions on data availability
- For clinical datasets or third party data, please ensure that the statement adheres to our policy

This Whole Genome project has been deposited at DDBJ/ENA/GenBank under the accession JBPKIN000000000 and the project number PRJNA1284617 and raw reads are available in the Sequence Read Archive (SRA) under accessions SRR37143892–SRR37143895. The version described in this paper is version JBPKIN010000000. The raw data of LC-MS analysis have been uploaded to Figshare https://doi.org/10.6084/m9.figshare.31458688 and at Science Data Bank https://doi.org/10.57760/sciencedb.27893.
The NCBI GenBank accession for SdCMT is PV857768. The NCBI GenBank accession for SdKSL1, SdKSL2, SdANS, SdHDAS, SdDAS, SdHDAO are PX964212-PX964218 .

Research involving human participants, their data, or biological material

Policy information about studies with human participants or human data. See also policy information about sex, gender (identity/presentation), and sexual orientation and race, ethnicity and racism.

Reporting on sex and gender	*Use the terms sex (biological attribute) and gender (shaped by social and cultural circumstances) carefully in order to avoid confusing both terms. Indicate if findings apply to only one sex or gender; describe whether sex and gender were considered in study design; whether sex and/or gender was determined based on self-reporting or assigned and methods used. Provide in the source data disaggregated sex and gender data, where this information has been collected, and if consent has been obtained for sharing of individual-level data; provide overall numbers in this Reporting Summary. Please state if this information has not been collected. Report sex- and gender-based analyses where performed, justify reasons for lack of sex- and gender-based analysis.*
Reporting on race, ethnicity, or other socially relevant groupings	*Please specify the socially constructed or socially relevant categorization variable(s) used in your manuscript and explain why they were used. Please note that such variables should not be used as proxies for other socially constructed/relevant variables (for example, race or ethnicity should not be used as a proxy for socioeconomic status). Provide clear definitions of the relevant terms used, how they were provided (by the participants/respondents, the researchers, or third parties), and the method(s) used to classify people into the different categories (e.g. self-report, census or administrative data, social media data, etc.) Please provide details about how you controlled for confounding variables in your analyses.*
Population characteristics	*Describe the covariate-relevant population characteristics of the human research participants (e.g. age, genotypic information, past and current diagnosis and treatment categories). If you filled out the behavioural & social sciences study design questions and have nothing to add here, write "See above."*
Recruitment	*Describe how participants were recruited. Outline any potential self-selection bias or other biases that may be present and how these are likely to impact results.*
Ethics oversight	*Identify the organization(s) that approved the study protocol.*

Note that full information on the approval of the study protocol must also be provided in the manuscript.

Field-specific reporting

Please select the one below that is the best fit for your research. If you are not sure, read the appropriate sections before making your selection.

[x] Life sciences [ ] Behavioural & social sciences [ ] Ecological, evolutionary & environmental sciences

For a reference copy of the document with all sections, see nature.com/documents/nr-reporting-summary-flat.pdf

Life sciences study design

All studies must disclose on these points even when the disclosure is negative.

Sample size	Prior determination of sample size was not a consideration for our data. Replicates of 3 and 3 were chosen for heterologous expression experiments in Saccharomyces cerevisiae strain AM119 and Nicotiana benthamiana respectively.
Data exclusions	No
Replication	For heterologous expression in Saccharomyces cerevisiae AM119, each experiment was tested 3 times. For heterologous expression in Nicotiana benthamiana, each experiment was tested 3 times.
Randomization	For heterologous expression in Saccharomyces cerevisiae AM119, each positive clone was randomized selected. For heterologous expression in Nicotiana benthamiana, each positive clone was randomized selected and the N. benthamiana plants were randomized infiltrated. Each of

	these plants would contain only one replicate from each different genes combination.
Blinding	Blinding was not relevant for this study. Functional characterization of genes or enzymes required the insight of researchers about the tested samples.

Reporting for specific materials, systems and methods

We require information from authors about some types of materials, experimental systems and methods used in many studies. Here, indicate whether each material, system or method listed is relevant to your study. If you are not sure if a list item applies to your research, read the appropriate section before selecting a response.

Materials & experimental systems

n/a	Involved in the study
☒	☐ Antibodies
☒	☐ Eukaryotic cell lines
☒	☐ Palaeontology and archaeology
☒	☐ Animals and other organisms
☒	☐ Clinical data
☒	☐ Dual use research of concern
☒	☐ Plants

Methods

n/a	Involved in the study
☒	☐ ChIP-seq
☐	☒ Flow cytometry
☒	☐ MRI-based neuroimaging

Plants

Seed stocks	*Report on the source of all seed stocks or other plant material used. If applicable, state the seed stock centre and catalogue number. If plant specimens were collected from the field, describe the collection location, date and sampling procedures.*
Novel plant genotypes	*Describe the methods by which all novel plant genotypes were produced. This includes those generated by transgenic approaches, gene editing, chemical/radiation-based mutagenesis and hybridization. For transgenic lines, describe the transformation method, the number of independent lines analyzed and the generation upon which experiments were performed. For gene-edited lines, describe the editor used, the endogenous sequence targeted for editing, the targeting guide RNA sequence (if applicable) and how the editor was applied.*
Authentication	*Describe any authentication procedures for each seed stock used or novel genotype generated. Describe any experiments used to assess the effect of a mutation and, where applicable, how potential secondary effects (e.g. second site T-DNA insertions, mosiacism, off-target gene editing) were examined.*

Flow Cytometry

Plots

Confirm that:

☒ The axis labels state the marker and fluorochrome used (e.g. CD4-FITC).

☒ The axis scales are clearly visible. Include numbers along axes only for bottom left plot of group (a 'group' is an analysis of identical markers).

☐ All plots are contour plots with outliers or pseudocolor plots.

☒ A numerical value for number of cells or percentage (with statistics) is provided.

Methodology

Sample preparation	About 0.2 g fresh Salvia divinorum leaves were cut and collected, chopped into small pieces with sharp blade and saturated in 500 L Nuclei Extraction buffer. After 60 s, the liquid buffer was filtered through a 50 m strainer and mixed with 2 mL staining buffer (Sysmex CyStainPI Absolute P kit) containing PI (propidium iodide) and RNase for 30 minutes in the dark.
Instrument	The suspension was analyzed by CyFlow Cube6 Flow Cytometer (Sysmex Partec, Muenster, Germany).
Software	The suspension was analyzed by FCSExpress software.
Cell population abundance	This section is not involved, as cell sorting was not performed; the flow cytometry conducted is for analysis.Gating based on the plot of FSC-SSC signal, gathered single cells area was gated for genome size analysis.
Gating strategy	Gating based on the plot of FSC-SSC signal, gathered single cells area was gated for genome size analysis.

☐ Tick this box to confirm that a figure exemplifying the gating strategy is provided in the Supplementary Information.
